# Medical cannabis use in Canada: vapourization and modes of delivery

**DOI:** 10.1186/s12954-016-0119-9

**Published:** 2016-10-29

**Authors:** Samantha Shiplo, Mark Asbridge, Scott T. Leatherdale, David Hammond

**Affiliations:** 1 School of Public Health & Health Systems, University of Waterloo, 200 University Ave W, Waterloo, ON N2L 3G1 Canada; 2 Centre for Clinical Research, Dalhousie University, Halifax, Canada

**Keywords:** Medical cannabis, Policy, Population health

## Abstract

**Background:**

The mode of medical cannabis delivery—whether cannabis is smoked, vapourized, or consumed orally—may have important implications for its therapeutic efficacy and health risks. However, there is very little evidence on current patterns of use among Canadian medical cannabis users, particularly with respect to modes of delivery. The current study examined modes of medical cannabis delivery following regulatory changes in 2014 governing how Canadians access medical cannabis.

**Methods:**

A total of 364 approved adult Canadian medical cannabis users completed an online cross-sectional survey between April and June 2015. The survey examined patterns of medical cannabis use, modes of delivery used, and reasons for use. Participants were recruited through a convenience sample from nine Health Canada licensed producers.

**Results:**

Using a vapourizer was the most popular mode of delivery for medical cannabis (53 %), followed by smoking a joint (47 %). The main reason for using a vapourizer was to reduce negative health consequences associated with smoking. A majority of current vapourizer users reported using a portable vapourizer (67.2 %), followed by a stationary vapourizer (41.7 %), and an e-cigarette or vape pen (19.3 %). Current use of a vapourizer was associated with fewer respiratory symptoms (AOR = 1.28, 95 % CI 1.05–1.56, *p* = 0.01).

**Conclusions:**

The findings suggest an increase in the popularity of vapourizers as the primary mode of delivery among approved medical users. Using vapourizers has the potential to prevent some of the adverse respiratory health consequences associated with smoking and may serve as an effective harm reduction method. Monitoring implications of such current and future changes to medical cannabis regulations may be beneficial to policymakers.

## Background

Cannabis has a range of therapeutic benefits including as an analgesic for pain, antispasm for multiple sclerosis, anticonvulsive for epilepsy, nausea suppressant for chemotherapy, and appetite stimulant for wasting in HIV/AIDS patients [1, 2]. Canadians were granted the legal right to access medical cannabis in 2001 following a ruling of the Supreme Court of Canada. In 2011, approximately 400,000 Canadians reported using cannabis for medical purposes [3]; however, only 40,000 Canadians had received approval to do so under Health Canada regulations as of 2013 [4, 5].

Medical cannabis can be delivered through various modes, including smoking, eaten in foods, vapourized, and in a spray. Although most medical cannabis users in Canada report trying multiple modes, smoking has been the dominant mode of delivery [6–9]. Canadian users report several advantages for smoked modes of delivery, including greater enjoyment, convenience and ease of use, more immediate and effective relief of symptoms, greater control over dosage, lower dose for desired effect, and whole-body euphoria [10, 11]. Data from other countries is consistent with Canadian studies, indicating greater use, preference, and cost-effectiveness of smoked delivery modes [6, 7].

Despite the appeal among users, smoked modes of delivery have several disadvantages. These include social disapproval for smoking and smell, as well as concerns about increased health risks from smoke inhalation [11, 12]. Studies have consistently shown that cannabis smokers report a higher frequency of cough and sputum production, wheezing, and bronchitis compared with non-smokers, as a result of airway inflammation and infection [13]. In addition, some literature reports the presence of lung cancer among heavy cannabis smokers, as well as bullous lung disease and emphysema [14]. Evidence on the long-term respiratory effects of cannabis smoking is complicated by the co-morbidity of cannabis and cigarette smoking (including mixing tobacco and cannabis), as well as time lag in the onset of chronic respiratory diseases [15, 16]. Nevertheless, chronic smoke inhalation from cannabis smoke is likely to reduce respiratory health.

Alternative modes of delivery have the potential to reduce the negative respiratory health risks associated with smoking cannabis. Cannabis can be consumed orally in edibles and oro-mucosally in sprays or tinctures [2]. Oral modes of delivery are perceived by users as healthier than smoking, less obvious than smoking since there is no smell, more convenient, and to have longer lasting effects [6, 7, 11]. On the other hand, medical cannabis users have reported that edibles do not provide the same euphoria, are more expensive, difficult to titrate dose and prepare, and have a slow onset of effect [6, 7, 11, 17].

Vapourization provides delivery characteristics that are similar to smoking, with respect to the time to onset and some sensory effects [6, 18]. However, vapourizers do not heat marijuana to the point of combustion and, therefore, expose users to significantly lower levels of toxicants that are only present in smoke [13]. Chemical analysis, self-reported data, and spirometry testing demonstrate that vapourization of cannabis is less harmful and reduces respiratory effects compared to smoking [13, 18–21]. Users perceive vapourization similar to smoking in terms of ease of dose titration and fast onset of action, but with fewer side effects [6, 22]. Vapourizing has also been reported to taste better, has no smoke smell, and is more discreet [6, 22]. For example, a recent international study among both medical and recreational users found that respondents reported their vapourizer experiences as satisfying or very satisfying and that 98 % of those respondents indicated that they intended to continue using a vapourizer [22]. Common disadvantages associated with vapourizers include greater inconvenience, the difficulty of using vapourizers, and the higher cost [22].

To date, there is little evidence in Canada on the prevalence of different modes of delivery. Although vapourizers have been on the market for some time, the rapid increase in the use of vapourizers to deliver nicotine (often referred to as “e-cigarettes”) has increased the availability of these products, while reducing their cost [23]. The extent to which these changes may have altered modes of delivery among medical cannabis users is unclear. In addition, regulatory changes in the way that approved users access medical cannabis in Canada may also have implications for common modes of delivery. Prior to April 2014, there were limited restrictions regarding which forms of medical cannabis were accessible to approved medical cannabis users. However, after the Marihuana for Medical Purposes Regulation (MMPR) was implemented in April 2014 by Health Canada, approved users could only legally access cannabis in dried herb form. Currently, there is no evidence regarding the extent to which alternative modes of delivery are being used by medical users or whether their perceptions regarding modes of delivery have changed.

The current study sought to examine medical cannabis use among a sample of approved adult medical users in Canada. In particular, the study assesses (1) the prevalence of different modes of delivery; (2) patterns of vapourizer use, including frequency, form of cannabis, and type; (3) perceptions of differing modes of delivery; and (4) the importance placed on various dimensions (e.g. time to onset of effect, symptom relief, cost, accessibility, duration of effect, level of harm) when selecting a mode of delivery.

## Methods

### Study design and protocol

An online cross-sectional survey was conducted from April 29 until June 8, 2015. No public sampling frame was available from which to sample approved medical cannabis users nor was there any reliable way of verifying approved status without disclosure of sensitive health care records. Therefore, the current study recruited approved users through licensed producers—the only legal source of medical cannabis in Canada. At the time of the survey, a total of 16 licensed producers who were registering clients in Canada were identified and approached to assist with study recruitment. Nine licensed producers agreed and sent their registered customers an email invitation with information about how to contact the study investigators. Eligible respondents were approved medical cannabis users, 18 years of age or older, and reported cannabis use in the past 30 days for health reasons. Eligible respondents were provided with a unique password via email to access the survey. Respondents who completed the survey received $10 as a thank you for completing the survey, provided via an electronic gift card or interact email payment. In order to protect confidentiality and to minimize social desirability bias, email addresses were the only personally identifying information collected from respondents. The study received approval from the Office of Research Ethics at the University of Waterloo.

### Survey measures

Survey measures were drawn from previous sources and adapted for the current study [4, 6, 7, 13, 24]. New and adapted measures underwent cognitive interviewing with approved medical cannabis users [25].

### Sociodemographics

Ethnicity was classified as *White* or *Non-white* (including South Asian, Chinese, Black, Filipino, Latin American, Arab, Southeast Asian, West Asian, Korean, Japanese, Aboriginal, or multi-racial). Education levels included *Low* (completion of high school or less), *Moderate* (technical/trade school, community college, or some university, but no degree), and *High* (university degree or more). Respondents were classified into five regions of Canada: *Atlantic* (New Brunswick, Newfoundland and Labrador, Nova Scotia, and Prince Edward Island), *Prairies* (Alberta, Manitoba, and Saskatchewan), *Ontario and Northern* (Ontario, Northwest Territories, and Nunavut) *Quebec*, and *British Columbia*. Personal income levels included *Low* ($0 to $40,000), *Middle* ($40,001 to $80,000), and *High* (more than $80,001). Cigarette smoking status was classified as not at all, occasionally, or daily.

### Medical cannabis use

The amount of cannabis used was examined by asking respondents to report on average how many grams of marijuana they use per day, per week, or per month. Respondents reported their frequency of cannabis use in the past 3 months as every day, almost every day, or less than almost every day, as well as number of uses per day. The main medical reason for cannabis use included five categories: *Pain relief* (chronic pain and fibromyalgia), *Mental health* (anxiety or nerves, depression, ADHD, bipolar, PTSD), *Central nervous system* (multiple sclerosis, spinal cord injury, and epilepsy), *Side effects* (nausea or vomiting and lack of appetite or weight loss), and *Other* in which a text box was provided for further explanations (other, glaucoma, cancer, insomnia).

Self-reported respiratory symptoms were examined using six previously adapted questions [13]. An index was used to represent the number of respiratory symptoms reported (0–6). Perceived harm from smoking cannabis was assessed as *Low* (not at all harmful to my health), *Moderate* (a little or somewhat harmful), and *High* (very or extremely harmful).

### Prevalence of modes of delivery

Respondents were asked to report *Ever*, *Current* (i.e. past 30 day use), and *Preferred* modes of delivery by selecting from a list, as follows: smoking a joint, smoking a blunt, smoking a pipe, smoking a bong or waterpipe, using a vapourizer, eating in foods or baked goods (e.g. cookies, candy), drinking (e.g. tea), taking a pill (e.g. Marinol®/dronabinol or Cesamet®/nabilone), using a spray (e.g. Sativex/nabiximols), and other. Respondents who currently used multiple modes of delivery were also asked to report the percentage of use for each mode of delivery they currently used. The use of alternative modes of delivery refers to all non-smoking modes of delivery (i.e. using a vapourizer, eating, drinking, taking a pill, using a spray, or other).

### Vapourizer use

Vapourizer awareness was ascertained by asking respondents whether they had ever heard of “vapourizing or vaping marijuana” before the study. Acceptability, harm of vapourizers, and patterns of use (i.e. form, frequency, and type), as well as reasons and barriers for using a vapourizer, were assessed.

### Perceptions and personal importance of dimensions by mode of delivery

Perceptions of modes of delivery were examined by asking respondents to rate 12 dimensions on a scale from 1 to 5 for the modes of delivery “smoking”, “using a vapourizer”, and “eating in foods”, separately (see Table 3 for the list of dimensions). Respondents were asked to rate the importance of reasons for selecting modes of delivery on a scale from 1 to 5 by answering, “How important are each of the factors to you in your choice of how to use marijuana”.

### Analysis

All analyses were conducted using SPSS, Version 22 (IBM, Illinois). Descriptive statistics (means, standard deviation, and proportions) are reported for all primary outcomes and covariates. Two logistic regression models examined correlates for the outcomes *Current* modes of delivery (0 = smoked only, 1 = alternative) and *Current* use of vapourizers (0 = non-current use of a vapourizer, 1 = current use of a vapourizer). A linear regression model also examined correlates for the outcome respiratory symptoms (ranged from 0 = no respiratory symptoms to 6 = severe respiratory symptoms). The following set of covariates were entered into all models: age, gender, ethnicity, education, income, main medical reason, and perception of harm of smoking, with the addition of respiratory symptoms and cigarette smoking status included as covariates in the *Current* modes of delivery model and respiratory symptoms in the *Current* use of vapourizer model. Adjusted odds ratios (AOR) and 95 % confidence intervals (95 % CI) are reported. In addition, an ANOVA was used to examine differences in perceptions of three modes of delivery.

## Results

### Sample characteristics

A total of 364 respondents completed the survey after deleting cases with missing information for age (*n* = 1), gender (*n* = 8), and incorrect responses to a data integrity question (*n* = 27). The survey’s completion rate (COMR) was 79.4 % using The American Association for Public Opinion Research standards [26].

Table 1 shows sample characteristics. As Table 1 indicates, more than half of the sample was male, approximately 75 % were “White”, and approximately half lived in Ontario. The most common medical reason was pain relief, and a majority of respondents use cannabis more than once a day.

**Table 1:** Sample characteristics

Characteristics	Overall	Current vapourizer use	No current vapourizer use
	(*N* = 364)	(*N* = 192)	(*N* = 102)
	Mean (SD)	Mean (SD)	Mean (SD)
Age (years)	40.8 (12.6)	41.2 (1.0)	44.3 (1.2)
	% (*n*)	% (*n*)	% (*n*)
Gender			
Male	57.7 (210)	67.2 (129)	52.9 (54)
Female	42.3 (154)	32.8 (63)	47.1 (48)
Ethnicity			
White only	74.7 (272)	86.5 (166)	78.4 (80)
Mixed/other/missing	25.3 (92)	13.5 (26)	21.6 (22)
Education			
Low	29.1 (106)	22.4 (43)	34.3 (35)
Moderate	46.7 (170)	49.5 (95)	49.0 (50)
High	22.0 (80)	27.1 (52)	16.7 (17)
Not reported	2.2 (8)	–	–
Income			
Low	58.5 (213)	56.8 (109)	63.7 (65)
Middle	18.1 (66)	19.8 (38)	17.6 (18)
High	14.0 (51)	17.7 (34)	9.8 (10)
Not reported	9.4 (34)	5.7 (11)	8.8 (9)
Region			
Ontario	50.3 (183)	64.1 (123)	46.1 (47)
British Columbia	15.1 (55)	15.1 (29)	18.6 (19)
Atlantic	13.2 (48)	13.0 (25)	8.8 (9)
Prairies	13.0 (47)	4.7 (9)	16.7 (17)
Quebec	4.1 (15)	2.6 (5)	5.9 (6)
Northern	3.8 (14)	–	2.9 (3)
Missing	0.5 (2)	0.5 (1)	1.0 (1)
Cigarette smoking status			
Not at all	58.5 (213)	76.7 (147)	59.8 (61)
Occasionally	16.2 (59)	6.3 (12)	8.8 (9)
Daily	19.2 (70)	17.2 (33)	30.4 (31)
Missing	6.1 (22)	–	1.0 (1)
Main medical reason			
Pain relief	44.8 (163)	57.8 (111)	51.0 (52)
Mental health	15.1 (55)	16.7 (32)	16.7 (17)
Central nervous system	9.9 (36)	8.9 (17)	8.8 (9)
Side effects	3.0 (11)	2.6 (5)	3.9 (4)
Other	12.4 (45)	13.0 (25)	14.7 (15)
Do not know	8.5 (31)	0.5 (1)	2.0 (2)
Refuse to answer	6.3 (23)	0.5 (1)	2.9 (3)
Amount (g/day)	1.8 (1.6)	1.8 (0.1)	1.6 (0.2)
Past 3-month frequency			
Less than almost every day	20.0 (73)	4.7 (9)	19.6 (20)
Almost every day	21.2 (77)	24.0 (46)	26.5 (27)
Every day	55.8 (203)	71.4 (137)	53.9 (55)
Do not know/refuse to answer	3.0 (11)	–	–
Number of times per day^a^			
Once	15.9 (58)	14.1 (27)	24.5 (25)
More than once	82.4 (300)	86.0 (165)	75.4 (77)
Do not know/refuse to answer	1.7 (6)	–	–

^a^Number of times per day represents the average number of times that medical cannabis was used per day on the days that medical cannabis was used in the past 30 days

### Prevalence of modes of delivery

Table 2 shows the prevalence of *Ever*, *Current*, and *Preferred* modes of delivery. The most common *Ever* mode of delivery tried was smoking a joint, whereas using a spray was the least common. Respondents reported *Ever* use of a mean of 5.4 (SD = 2.2) modes of delivery.

**Table 2:** Prevalence of modes of delivery (*N* = 364)

Mode	Ever use	Current use	Preferred
	% (*n*)	% (*n*)	% (*n*)
Smoked modes			
Smoking a joint	74.2 (270)	47.0 (171)	23.1 (84)
Smoking a pipe	61.3 (223)	25.8 (94)	4.9 (18)
Smoking a bong/waterpipe	53.6 (195)	21.4 (78)	8.5 (31)
Smoking a blunt	38.7 (141)	4.7 (17)	1.1 (4)
*At least one smoked*	*77.2 (281)*	*58.8 (214)*	*37.6 (137)*
Alternative modes			
Using a vapourizer	65.9 (240)	52.7 (192)	28.3 (103)
Eating in foods	64.1 (234)	31.0 (113)	7.1 (26)
Drinking	29.9 (109)	5.5 (20)	1.6 (6)
Taking a pill	28.6 (104)	3.3 (12)	0.8 (3)
Using a spray	8.2 (30)	1.1 (4)	0.3 (1)
Other	10.4 (38)	8.0 (29)	4.7 (17)
*At least one alternative*	*76.1 (277)*	*63.7 (232)*	*42.9 (156)*
Do not know	9.6 (35)	9.6 (35)	10.4 (38)
Refuse to answer	9.6 (35)	9.9 (36)	9.9 (36)

The most common *Current* and *Preferred* mode of delivery was a vapourizer (see Table 2). Respondents reported *Current* use of a mean of 2.4 (SD = 1.3) modes of delivery. Among those who reported multiple modes, smoking a joint was reported 53.1 % (SD = 36.8) of the time, followed by 52.4 % (SD = 36.4) using a vapourizer, 43.5 % (SD = 39.6) other, 36.1 % (SD = 31.4) smoking a bong or waterpipe, 31.6 % (SD = 31.8) smoking a pipe, 26.4 % (SD = 25.8) taking a pill, 25.0 % (SD = 29.3) eating in foods or baked goods, 22.6 % (SD = 29.0) drinking, 20.0 % (SD = 10.8) using a spray, and 13.5 % (SD = 13.4) smoking a blunt.

Table 1 also shows sample characteristics stratified among participants who reported current use of a vapourizer and those who did not report current use of a vapourizer. A logistic regression model (not shown) was fitted to examine factors associated with *Current* modes of delivery. Respondents with a high level of education had an increased odds of currently using an alternative mode of delivery compared to those with a low level of education (AOR = 4.92, 95 % CI 1.44–16.84, *p* = 0.01) and those with a moderate level of education (AOR = 5.00, 95 % CI 1.43–16.67, *p* = 0.01). Respondents who used medical cannabis mainly for “other” reasons had an increased odds of using alternative modes of delivery compared to those who used for pain relief, poor mental health, and central nervous system illnesses (AOR = 3.41, 95 % CI 1.08–10.70, *p* = 0.04; AOR = 9.09, 95 % CI 1.96–33.33, *p* = 0.01; and AOR = 16.67, 95 % CI 2.04–100.00, *p* = 0.01, respectively). Respondents who used medical cannabis for relief of side effects had increased odds of using alternative modes of delivery compared to those using for poor mental health and central nervous system illnesses (AOR = 8.91, 95 % CI 1.05–75.72, *p* = 0.05 and AOR = 17.54, 95 % CI 1.33–231.28, *p* = 0.03, respectively).

Respondents who perceived higher levels of harm from smoking cannabis had an increased odds of currently using alternative modes of delivery relative to those who perceived the harm from smoking cannabis as “low” (AOR = 16.90, 95 % CI 3.88–73.62, *p* < 0.01) or “moderate” (AOR = 16.67, 95 % CI 2.13–100.00, *p* < 0.01). Those who reported having a lower number of respiratory symptoms had a higher odds of currently using alternative modes of delivery (AOR = 1.61, 95 % CI 1.16–2.17, *p* < 0.01). In terms of smoking cigarettes, non-smokers or “occasional” smokers also had higher odds of using alternative modes of delivery compared to daily smokers (AOR = 6.25, 95 % CI 1.56–25.00, *p* = 0.01 and AOR = 6.23, 95 % CI 0.38–23.83, *p* = 0.01, respectively). No other significant differences were observed by gender, age, ethnicity, income, or region.

### Vapourizer use

Overall, 77.2 % (*n* = 281) of respondents were aware of vapourizing or vaping prior to the study, 11.5 % (*n* = 42) of respondents were not aware of vapourizing or vaping before the study, 3.3 % (*n* = 12) of respondents did not know, and 8.0 % (*n* = 29) of respondents refused to answer. A total of 71.6 % reported trying a vapourizer; among those individuals, 28.3 % reported daily vapourizer use over the past 30 days, 18.3 % used a vapourizer almost every day over the past 30 days, 14.2 % used a vapourizer at least once a week in the past 30 days, 19.2 % used a vapourizer at least once in the last 30 days, and 20.0 % had used a vapourizer, but not in the past month.

Respondents were asked to report the use of different forms of cannabis in a vapourizer. Among those who *Ever* used a vapourizer, virtually all had used dried herb in a vapourizer (97.5 %), followed by cannabis resin (19.6 %), butane extract (18.8 %), oil (14.6 %), and an alcohol or carbon dioxide extract (1.7 %). Current users reported similar proportions of using different forms (data not shown). With respect to the type of vapourizer used, a majority of current users reported using a portable vapourizer (67.2 %), followed by a stationary vapourizer 41.7 %, and an e-cigarette or vape pen (19.3 %).

A logistic regression model (not shown) was fitted to examine factors associated with the *Current* use of vapourizers. Males (AOR = 2.46, 95 % CI 1.30–4.76, *p* < 0.01), younger age (AOR = 1.03, 95 % CI 1.00–1.05, *p* = 0.05), “white” ethnicity (AOR = 2.52, 95 % CI 1.03–6.19, *p* = 0.04), and those with fewer respiratory symptoms (AOR = 1.28, 95 % CI 1.05–1.56, p = 0.01) reported a higher odds of *Current* use of vapourizers. In addition, significant differences were observed between regions (*p* = 0.02): respondents from the Prairie Provinces (AOR = 5.69, 95 % CI 1.50–21.50, *p* = 0.01) and from Ontario and Northern regions (AOR = 5.29, 95 % CI 1.73–16.19, *p* < 0.01) had a higher odds of currently using a vapourizer than those from the Atlantic Canadian Provinces. No significant differences were observed for education, income, main medical reason for using cannabis, and perceived harm of cannabis smoking.

A regression model was conducted to examine the association between vapourizer use and respiratory symptoms in more detail. Using a vapourizer was significantly associated with lower respiratory symptoms (*B* = −0.40, *p* = 0.049) after adjusting for cigarette smoking status, as well as age, gender, ethnicity, education, income, and main medical reason for cannabis use.

### Perceptions of modes of delivery

Respondents were asked to rate three modes of delivery—smoking, using a vapourizer, and eating in food—along 12 different dimensions using a scale range from 1 to 5 for each dimension. Table 3 shows the mean rating by mode of delivery for each of the 12 dimensions. ANOVA models were run to test for differences in the mean rating between the three modes, for each of the 12 dimensions. As indicated by the superscript letters in Table 3, time to onset of effect was rated as significantly faster for smoking, followed by using a vapourizer, and then eating in food. Smoking was rated as significantly lower cost and more accessible than using a vapourizer or eating in food. However, using a vapourizer and eating in food was rated as having a lower level of perceived harm than smoking. Using a vapourizer was rated as easiest to use, and the number of side effects was rated significantly lower for using a vapourizer compared to smoking or eating in food. Eating in foods was rated as producing the worst high, most stigma, was the hardest to find a correct dose, but had the longest duration of effect. Lastly, perceptions of symptom relief did not differ by mode.

**Table 3:** Perceptions of modes of delivery by dimension (*n* = 342)

Dimensions	Smoking	Using a vapourizer	Eating in food
	Mean (SD)	Mean (SD)	Mean (SD)
Time to onset of effect	4.1 (1.0)a	3.8 (1.1)a	2.1 (1.2)a
Symptom relief	4.0 (0.9)a	3.9 (1.0)b	3.9 (1.2)c
Accessibility	3.9 (1.2)a	3.6 (1.2)a	3.1 (1.4)a
Type of “high”	3.8 (1.0)a	3.8 (1.0)b	3.6 (1.1)ab
Ease of use	3.7 (1.2)a	3.8 (1.1)b	3.5 (1.4)b
Ability to find correct dose	3.7 (1.2)a	3.7 (1.2)b	2.6 (1.3)ab
Number of side effects	3.6 (1.2)a	4.0 (1.1)ab	3.7 (1.2)b
Duration of effect	3.4 (0.9)a	3.3 (1.0)b	4.2 (1.0)ab
Level of harm	3.3 (1.3)ab	4.2 (1.0)a	4.2 (1.1)b
Amount needed for effect	3.1 (1.0)a	3.2 (1.2)b	2.4 (1.2)ab
Cost	2.4 (1.2)ab	2.2 (1.2)a	2.2 (1.1)b
Stigma	2.4 (1.3)a	2.9 (1.3)a	3.3 (1.3)a

Rating was completed on a scale from 1 to 5, where 1 represented the least desired effect and 5 represented the most desired effect. Values with the same letter (a, b, c) are significantly different at a *p* < 0.05 tested using an ANOVA

### Perceptions of vapourizer use

Among respondents who currently used a vapourizer (*n* = 192), reported reasons for current vapourizer use were they are less harmful to me than smoking cannabis (79.7 %, *n* = 153), it does not smell as much as smoking cannabis (71.4 %, *n* = 137), easy to use (58.9 %, *n* = 113), uses less cannabis (56.8 %, *n* = 109), less harmful to people around me than smoking (51.0 %, *n* = 98), less side effects (40.1 %, *n* = 77), easy to find the correct dose (39.6 %, *n* = 76), provides the best symptom relief (34.4 %, *n* = 66), effects occur faster (27.1 %, *n* = 52), effects last longer (23.4 %, *n* = 45), provides the best high (20.8 %, *n* = 40), more affordable (16.7 %, *n* = 32), more accessible (15.6 %, *n* = 30), other people I know use a vapourizer too (11.5 %, *n* = 22), it is fun to use (11.5 %, *n* = 22), and other (9.4 %, *n* = 18).

Respondents who were aware of vapourizers, but had never used them (*n* = 41), were asked to report their main reason for not using a vapourizer. A total of 26.8 % reported they were simply not interested in using a vapourizer, followed by concerns about affordability (19.5 %) and difficulty using a vapourizer (9.8 %). Overall, 73.2 % of never users indicated they would be willing to try a vapourizer in the future.

Respondents who were aware of vapourization were asked about their perceptions regarding the acceptability and harm of vapourizing. As Table 4 shows, a majority of respondents felt that vapourizers were more acceptable to use compared to smoking, while a strong majority reported that vapourizers were less harmful than smoking. Almost half reported that vapourizing is “not at all harmful”.

**Table 4:** Perceptions of vapourization among those who are aware of vapourization (*n* = 279)

Perceptions	% (*n*)
Relative acceptability of vapourizers	
Less acceptable than smoking	5.4 (15)
As acceptable as smoking	27.2 (76)
More acceptable than smoking	59.1 (165)
Do not know	0.4 (1)
Refuse to answer	7.9 (22)
Harm of vapourizing	
Not at all harmful	48.0 (134)
A little or somewhat harmful	38.7 (108)
Very or extremely harmful	0.7 (2)
Do not know	12.2 (34)
Refuse	0.4 (1)
Relative harm of vapourizing	
Less harmful than smoking	82.8 (231)
As harmful as smoking	6.5 (18)
More harmful than smoking	1.1 (3)
Do not know	8.9 (25)
Refuse to answer	0.7 (2)

### Personal importance of dimensions by mode of delivery

Respondents were asked to rate the importance of each dimension in their choice of how to use cannabis. As indicated in Table 5, symptom relief was rated as the most important dimension when selecting the mode of delivery, followed by cost, whereas the least important dimension was stigma.

**Table 5:** Important dimensions for selecting modes of delivery (*N* = 364)

Dimensions	Rating of importance
	Mean (SD)
Symptom relief	4.5 (0.9)
Cost	4.2 (1.0)
Duration of effect	4.1 (1.0)
Accessibility	4.0 (1.1)
Amount needed for effect	4.0 (1.1)
Time to onset of effect	4.0 (1.1)
Ability to find correct dose	4.0 (1.1)
Ease of use	3.9 (1.1)
Type of “high”	3.8 (1.2)
Number of side effects	3.7 (1.3)
Level of harm	3.6 (1.4)
Stigma	2.6 (1.4)

Rating was completed on a scale from 1 to 5, where 1 represented not important at all and 5 represented extremely important

## Discussion

The current study provides the most comprehensive assessment of consumer perceptions and use of delivery modes among medical cannabis users to date. The findings suggest a possible shift in the popularity of modes of delivery among approved medical users. Using a vapourizer was the most common and the most preferred mode of delivery. Overall, alternative modes were slightly more popular than smoking, even though the MMPR restricted the use and sale of medical cannabis to only dried herb, which is most easily smoked. Previous studies of medical users all found that smoking was the most commonly used and most preferred mode, in contrast to the current study’s findings [7–9, 27–30]. Among past studies that examined vapourizer use, the prevalence of using a vapourizer was generally low, falling between 8 and 20 % [7, 28, 29]. However, one Canadian study, conducted in 2007, found that 88 % of approved medical users smoked, 72 % ate it, and 52 % used a vapourizer, which offers similar estimates to the current study [27]. Overall, the findings indicate a substantial shift towards the use of vapourizers and alternative modes of delivery among a sample of approved Canadian medical cannabis users.

The popularity of vapourizers for using cannabis may have been influenced by the recent increase in awareness and use of e-cigarettes, which vapourize nicotine [23]. Most of the respondents who used a vapourizer reported using a portable type of vapourizer, which is similar in style to an e-cigarette. The findings suggest a shift away from “stationary” vapourizers, such as the Volcano, which was reported as the most commonly used vapourizer in two previous studies among both medical and recreational cannabis users [6, 22]. Unfortunately, “stationary” vapourizers have better temperature control to ensure no combustion is occurring whereas “portable” vapourizers or vape pens are more likely to be heated quickly to a pre-set temperature using a battery, which can increase the likelihood for combustion possibly resulting in increased levels of toxicants [20, 31].

Findings from the current study add to the previous literature showing current use of alternative modes of delivery and current use of a vapourizer are associated with reporting fewer respiratory symptoms [13, 19, 32]. Using non-combustible modes could prevent some of the negative respiratory health consequences associated with smoking and may serve as an effective harm reduction method for the delivery of therapeutic relief through medical cannabis. Respondents perceived smoking and vapourizing as fairly similar on the 12 dimensions, with the exception that smoking was perceived as significantly more harmful, while vapourizing was associated with the fewest side effects. These findings suggest that most medical cannabis users who use a vapourizer do so because they are aware and understand that using a vapourizer reduces the negative health consequences linked with smoking. General concerns about smoking-related health consequences have also been noted by respondents in previous research [6, 7]. The current rise in health promotion, in terms of tobacco and smoking prevention legislation at all levels of government, most likely has also influenced the increase in vapourizer use as opposed to smoking.

Respondents did identify several perceived advantages to smoking; most notably, it provides the fastest time to onset of effects. However, past scientific data reported using a vapourizer and smoking produced similar blood cannabinoid concentration levels over the same time frame [2, 18]. Future research should examine the delivery kinetics of vapourizing versus smoking given the importance to users and, potentially, the therapeutic benefits of medical cannabis. Indeed, symptom relief was rated as the most important dimension when deciding which mode of delivery to use, suggesting that approved medical users prefer the mode that relieves their symptoms most effectively. However, respondents’ perceptions of the modes did not differ on symptom relief, indicating that each mode of delivery—smoking, using a vapourizer, and eating in foods—provided similar symptom relief. Given the range of medical reasons by which approved users have obtained approval, future research should explore whether particular modes of delivery are better suited for specific types of symptom relief. For example, acute pain relief may benefit from modes with faster onset, whereas chronic conditions may benefit from modes with longer duration of effect. These principles are well established with respect to modes of delivery for pharmaceutical drugs but have yet to be explored for medical cannabis use.

Finally, cost was cited as the second most important dimension when selecting delivery modes. Eating in foods and using a vapourizer were both perceived as the most expensive, and smoking the least expensive mode. Previous studies have also cited cost as a main reason for not using a vapourizer [22, 33]. For example, one study reported a mean cost of approximately $250 to purchase a vapourizer [22]. Cost may be important to medical cannabis users as cost coverage for medical cannabis use in general is low in Canada [34]. However, given the rise in popularity of the use of e-cigarette devices, the cost associated with vapourizing technology is dropping and portable devices for vapourizing similar to e-cigarettes are much less expensive compared to stationary vapourizers.

### Strengths and limitations

The current study has a number of strengths and limitations. A convenience sample was recruited through licensed producers as probability sampling was not feasible. Only approved medical cannabis users were eligible to take part in the survey, thus excluding medical users who were not approved. There is no way to determine whether the current sample is the representative of the population of approved medical cannabis users in Canada due to a lack of publicly accessible information on this population. However, in 2013, Health Canada released information showing that the majority of Canadians with medical cannabis approval resided in British Columbia, accounting for nearly half of all licensed medical users in Canada, followed by Ontario at approximately 30 % [5]. Therefore, the current sample may have potentially under represented British Columbia residents and over represented Ontario residents. Additional research on the profile of medical cannabis users in Canada would be an asset, particularly with respect to comparisons between approved and non-approved users. Finally, the survey relied on self-report, which may contribute to a number of biases, including recall bias and social desirability bias. For example, about a quarter of participants responded “I don't know”, “Other”, or “Refuse to answer” when asked what their main reason for medical cannabis use was. In addition, the survey was cross-sectional; thus, a temporal order could not be established.

Strengths of the study include a systematic recruitment of a large sample of approved medical cannabis users from across Canada. This is one of the first studies to only include approved Canadian medical cannabis users, which is the population directly impacted by medical cannabis regulations. The timeliness of the data was also a major strength as it was important to assess the possible implications of the MMPR, which to our knowledge had not been examined until the current study.

## Conclusions

This study presents a general picture of the current state of medical cannabis use among a sample of approved users in Canada. The findings indicate that approved users have tried multiple modes of delivery, but using a vapourizer was the most commonly used and most preferred mode. The findings suggest that increasing availability and lowering the cost of vapourizers is likely to reduce the prevalence of smoking medical cannabis even further, with the added benefits of reducing harm linked to smoking. Currently, it is unclear what, if any, guidance is being provided to medical cannabis users from physicians, health authorities, or licensed producers with respect to modes of delivery. More generally, the findings highlight the importance of monitoring patterns of medical cannabis use to a greater extent. For example, a Quebec Cannabis registry has been created in order to track patient use information, as well as monitor patient safety; national registries based on this model have the potential to inform medical cannabis policy, particularly given recent changes in federal regulation.
